# Differences in alcohol and cannabis use amongst substance use disorder patients with and without comorbid attention-deficit/hyperactivity disorder

**DOI:** 10.4102/sajpsychiatry.v28i0.1786

**Published:** 2022-04-14

**Authors:** Corné Coetzee, Ilse Truter, Anneke Meyer

**Affiliations:** 1 Drug Utilization Research Unit (DURU), Faculty of Health Sciences, Nelson Mandela University, Gqeberha, South Africa; 2 Department of Pharmacy, Faculty of Health Sciences, University of Limpopo, Sovenga, South Africa; 3 Drug Utilization Research Unit (DURU), Department of Pharmacy, Faculty of Health Sciences, Nelson Mandela University, Gqeberha, South Africa

**Keywords:** ADHD, alcohol, ASRS, cannabis, DIVA, self-medication, South Africa

## Abstract

**Background:**

Substance use disorders (SUDs) continue to be a public health problem. Attention-deficit/hyperactivity disorder (ADHD) is seen as a risk factor for SUD. Prevalence of alcohol and cannabis use amongst adults with SUD and comorbid ADHD impacts both disorders cognitively and behaviourally.

**Aim:**

Our study aimed to compare alcohol and cannabis use between treatment-seeking SUD patients with ADHD and SUD patients without ADHD symptomatology.

**Setting:**

Various rehabilitation centres, including the South African National Council on Alcoholism and Drug Dependence (SANCA) Centres, and Private and Provincial Government Substance Abuse Treatment Centres.

**Methods:**

A cross-sectional study of adults on drug rehabilitation was conducted. Data on socio-demographic information and alcohol and cannabis use from 185 post-detox inpatients were collected. Diagnoses were based on DSM-IV criteria. Attention-deficit/hyperactivity disorder diagnosis was confirmed by the Diagnostic-Interview for ADHD in Adults (DIVA 2.0). SUD+ADHD (*n* = 52) and SUD-ADHD (*n* = 128) groups were compared on alcohol and cannabis use as a function of gender.

**Results:**

No significant differences in the use of alcohol between the SUD+ADHD and SUD-ADHD groups were found. However, the SUD+ADHD group showed increased cannabis consumption. Especially, the SUD+ADHD females showed an earlier age of onset of cannabis use than the SUD-ADHD females and revealed that they use cannabis for a longer period compared with the SUD-ADHD females and SUD+ADHD and SUD-ADHD males.

**Conclusion:**

The results revealed the relationship between ADHD and cannabis use, especially amongst females with ADHD and reinforce the need to consider ADHD in cannabis use SUD in clinical interventions.

## Introduction

Attention-deficit/hyperactivity disorder (ADHD) is a common childhood neurodevelopmental disorder characterised by early onset of impairing levels of hyperactivity, inattention and impulsiveness.^1^ Symptoms persist into adulthood for a considerable proportion of patients.^2,3^

Attention-deficit/hyperactivity disorder has a worldwide prevalence in the general population of 3.4%.^4^ Up to 65% of children with ADHD continue to have symptoms and related impairment persisitent in adulthood.^3^ Even though the prevalence is shown to decrease when patients with ADHD reach adulthood, this suggests that symptoms are still present in a significant number of females and males, albeit they may manifest differently and can therefore be more difficult to detect.^5^

Whereas hyperactivity symptoms commonly remit over time, impulsiveness and attentional deficits more often persist into adulthood.^6^ In adult ADHD, hyperactivity symptoms often present as inner restiveness as opposed to motor activity. Similarly, in adults, impulsiveness more often manifests in unsatisfactory occupational performance,^7,8^ drug abuse^8^ and other disadvantageous behaviours.^9^

It is widely reported that several mental health disorders are overrepresented amongst substance abuse populations.^10,11^ Substance use disorder (SUD) is a frequent comorbidity in adolescents and adults with ADHD.^12,13,14,15,16^ ADHD is highly prevalent in individuals seeking treatment for SUD,^17,18,19^ with between 8% and 46% of those diagnosed with ADHD are reported to have comorbid SUD.^10,19^

Numerous studies have shown an increased risk of developing substance use and SUDs, including alcohol, tobacco and illicit drugs, in patients with ADHD.^20,21,22,23^ Several mechanisms might be involved in the increased risk of SUD in ADHD. Neurobiological models indicate an imbalance in the dopaminergic neurotransmission, in both disorders.^24,25^ The mesolimbic dopamine (DA) pathway originally seemed to be crucial for drug reward.^26^ However, other DA pathways, nigrostriatal and mesocortical, also contribute to drug reward and addiction.^27^ Wise concluded that both these pathways participated in reward function and addiction. Electrical stimulation of both these pathways is rewarding. Blockade of glutamatergic or cholinergic input to either of the pathways attenuates the habit-forming effects of intravenous cocaine and dopamine in both nigrostriatal and mesocorticolimbic pathways.^26,27^

The self-medication hypothesis (SMH) has been used to explain substance use because drugs of abuse reduce psychological distress.^28^ Another aspect of the SMH is that different disorders are associated with specific types of illicit drugs because of pharmacological specificity.^29^ When individuals with a disorder, experiment with various illicit drugs, they will find that certain drugs relieve emotional or affective states that are causing them problems. The SMH considers that psychiatric disorders precede substance abuse. However, it is not the psychiatric diagnosis that is self-medicated; instead, it is the psychological suffering described by the diagnosis.^30^

Alcohol is one of the most widespread substances of abuse. Reports showed that South Africa had experienced a remarkable increase in the use of various substances of abuse.^31^ Although alcohol is still frequently used as a substance of abuse in South Africa, the latest data from the South African Community Epidemiology Network on Drug Use (SACENDU) shows that cannabis is currently the most widely used drug in South Africa. Amongst those seeking professional treatment at rehabilitation facilities, there was a significant increase in the proportion of cannabis admissions from less than 1.9% of admissions in 2000 to 8.3% in 2013.^32^

Cannabis, also known as dagga, has been an illegal substance in most parts of the world because of its deleterious nature. Historically, strict rules governed the use of cannabis. However, the change is evolving faster as several countries and nations have or are revising regulations to soften regulated and prohibited laws against its use and production; thereby leaving vulnerable individuals more prone at the receiving end.^33^ The use of cannabis is now more accessible to younger and poly-users in the context of modernised, increasingly urbanised societies where old-fashioned controls are collapsing. South Africa is a substantial producer of cannabis (the world’s third largest), most of which is consumed in the southern African region.^33^ Cannabis is considered by the World Health Organization (WHO) (2015)^34^ as a drug that is commonly cultivated, trafficked and consumed in the world. This means that its availability to people will continue effortlessly. Therefore, it is reported that this affordability and availability of cannabis is also an important factor influencing substance use by emerging adults, as a strong correlation between the prevalence of cannabis use has been reported throughout life and the feeling of easy availability.^35^

The risk of SUD is significantly higher in people with ADHD compared with those who are without ADHD.^23,36,37^ Some data have significantly suggested the role of ‘self-medication’ as a mechanism for the development of SUD. For example, there have been reports of the ‘calming effects’ of alcohol and cannabis amongst individuals with ADHD.^28,38^ When not treated sufficiently, individuals with ADHD may use psychoactive substances to self-manage and curtail ADHD symptoms or to express their impulsiveness.^39^ Literature has shown that early detection of ADHD plays a significant role in the development of SUD in such individuals. Furthermore, past pharmacotherapy protects ADHD individuals from self-medicating and developing SUD and significantly improves ADHD-related impairment across all domains.^23,30,37,40,41^ In the past decade, new non-pharmacologic interventions have been developed, such as working memory, intensive school-based interventions for adolescents and neurofeedback and parental training.^42^ For this study non-pharmacological interventions were not taken into account.

In terms of the prevalence of ADHD symptomology in adults and its clinical characteristics, ADHD is more common amongst patients who use cannabis than amongst patients seeking other SUD treatments. In addition, self-medication may not be specific for ADHD symptoms, but rather a way to alleviate co-occurring symptoms related to mood and anxiety, which is common in ADHD patients.^43,44^

The aim of our study, therefore, was to compare alcohol and cannabis use between a group of treatment-seeking SUD patients with and without ADHD.

## Research methods and design

### Study design

A quantitative, cross-sectional, case-control study of adults seeking treatment for substance abuse was employed.

### Setting

Our study was conducted at various rehabilitation centres, including Private and Provincial Government Substance Abuse Treatment Centres, the South African National Council on Alcoholism and Drug Dependence (SANCA) Help Centres and psychiatric hospitals in South Africa.

### Study population and sampling

A convenience sample was used. Altogether, data from 185 post-detox inpatients on drug rehabilitation were collected from seven facilities from April 2018 to November 2019. Facilities were chosen within driving vicinity of researchers involved in our study. Participants were over 18 years of age, seeking treatment as inpatients in the facilities, who had completed primary school to ensure sufficient command of the English language. Exclusion criteria were a history of head injuries and severe neurological disorders, that is, epilepsy, cerebral palsy and severe psychiatric disorders.

### Instruments

#### Sociodemographic information

A questionnaire containing general demographical considerations including ethnicity, home language, education level and employment status.

#### Alcohol and cannabis use information

A comprehensive list of a various addictive substances to examine historical or present (active within the month prior to admission) use of substances and start age was completed by the participants.

#### Adult ADHD Self-Report Scale (ASRS v.1)

The ASRS is designed to detect ADHD symptoms in adulthood by relating symptoms to adult situations such as projects, tasks or work. This reliable and valid self-administered instrument was jointly developed in 2005 by the WHO and Kessler and colleagues.^45^ It consists of 18 questions based on the criteria diagnosing ADHD according to DSM-IV-Text Revision. The first six questions (ASRS-6) are most predictive of symptoms consistent with a diagnosis of ADHD.^45,46^ Participants were expected to rate the frequency of symptoms that they experienced on a five-point Likert-type scale: never (0), rarely (1), sometimes (2), often (3) and very often (4).

Cronbach’s α was computed for the entire sample at 0.85 for inattention and at 0.83 for hypersensitivity-impulsiveness.

#### Diagnostic Interview for ADHD in adults (DIVA 2.0)

The DIVA 2.0 is a structured interview for ADHD in adults based on the DSM-IV-TR criteria. The instrument was developed by Kooij and Francken and is the successor of the initial semi-structured interview for ADHD in Adults.^47,48^ It is divided into two domains to evaluate symptoms applicable to childhood (before age 12) and adulthood, whilst the third part considers functional impairment in five areas of functioning in both age categories.^49^ To simplify the evaluation of each of the 18 symptom criteria for ADHD, the interview provides a list of specific and realistic examples for both current and retrospective childhood behaviour. The examples are based on conventional descriptions provided by adult patients in clinical practice. Examples are also provided for the types of impairment that are typically associated with the five areas of everyday life: relationships and family life, work and education, social contacts, free time and hobbies, self-confidence and self-image.

### Data collection

The data collection consisted of two stages.

#### Screening

Our study was presented and explained to participants in English, and after their written consent, a questionnaire (demographic information, substance use information and ASRS) was distributed. The authors distributed all questionnaires and were available for questions if needed. The response rate for the questionnaires was 99%.

#### A diagnostic interview

All participants who screened positive for ADHD based on their scores on the ASRS-6 (cut-off score for the first six questions of the ASRS was 16) were interviewed making use of the DIVA 2.0 to confirm the presence of ADHD. They formed the SUD+ADHD group. The participants who screened negative on the ASRS-6 formed the SUD-ADHD group.

#### Data analysis

Data from the Demographic Information and Substance Use questionnaires were entered and analysed using Statistica v. 12 (Dell Software). Multivariate analysis of variance (MANOVA) was employed to establish differences in alcohol and cannabis use, using a 2 × 2 (SUD+ADHD vs. SUD-ADHD x gender) model. Differences in categorical data (demographics) were established by *X*^2^ analysis. The level of statistical significance was set at *p* = 0.05.

### Ethical considerations

Our study was conducted in accordance with the 1964 Declaration of Helsinki^50^ and was approved in agreement with the ethical requirements of the Research Ethics Committee (Human) of Nelson Mandela University (no. H14-HEA-PHA-007).

Each rehabilitation facility’s respective management provided permission to perform our study at the involved centres. The participants were provided with an information document describing the investigation process. Written informed consent was obtained from the participants before the start of completing the questionnaires. The option of withdrawing at any stage of the procedure was given.

## Results

Of the 185 participants who were screened for ADHD, 57 screened positive for the disorder using the ASRS-6. Subsequent confirmation of ADHD symptomatology using the DIVA established ADHD in 52 participants. The final sample, therefore, consisted of 180 participants, 52 (29%) classified as SUD+ADHD and 128 (71%) as SUD-ADHD (see Table 1).

**TABLE 1:** Demographics of the sample.

Demographic variable	SUD+ADHD *N* = 52 (29%)		SUD-ADHD *N* = 128 (71%)		*p*	Effect size
	*n*	%	*n*	%		
**Age**					0.93	0.03
18–29	23	44.2	60	46.9		
30–40	19	36.6	46	35.9		
41+	10	19.2	22	17.2		
**Gender**					0.06	−0.14
Male	35	67.3	103	80.5		
Female	17	32.7	25	19.5		
**Ethnicity**					0.002*	0.33
African	14	26.9	77	60.2		
Mixed race	10	19.2	15	11.7		
White	28	53.9	33	25.8		
Indian	0	0.0	3	2.3		
**Home language**					0.05*	0.32
Afrikaans	24	46.2	30	23.4		
English	13	25.0	21	16.4		
Sepedi	3	5.8	7	5.5		
Xitsonga	1	1.9	2	1.6		
Tshivenda	0	0.0	3	2.3		
isiXhosa	5	9.6	25	19.5		
isiZulu	2	3.8	16	12.5		
Setswana	3	5.8	10	7.8		
Sesotho	1	1.9	7	5.5		
siSwati	0	0.0	2	1.6		
isiNdebele	0	0.0	5	3.9		
**Employment**					0.39	0.19
Employed	26	50.0	52	40.6		
Unemployed	17	32.7	54	42.3		
Part-time	3	5.8	4	3.1		
Self employed	0	0.0	3	2.3		
Student	6	11.5	10	7.8		
**Pensioner**	0	0.0	4	3.1		
**Other**	0	0.0	1	0.8		
**Education level**					0.004*	0.27
Primary completed	0	0.0	3	2.3		
Grade 8–11	9	17.3	56	43.8		
Secondary completed	24	46.2	36	28.1		
Tertiary	19	36.5	33	25.8		

SUD, Substance use disorder; ADHD, Attention-deficit/hyperactivity disorder.

* , *p* ≤ 0.05.

No significant differences in age or distribution between the two groups were found. For both the SUD+ADHD and SUD-ADHD groups, the females were in the minority. Although not statistically significant, there were relatively more females in the ADHD group. For both groups, most participants came from the 18–29 age group. There was a significant difference in ethnic representation (*p* = 0.002), with significantly more white people (53.9%) amongst the ADHD group when compared with the non-ADHD group (25.8%). Also, a significant difference in language group representation was found, with a higher prevalence of Afrikaans speakers in the SUD+ADHD group (46.2%) than in the SUD-ADHD group (23.4%). A comparison of the employment status showed no significant difference, but the ADHD group showed a significantly higher level of education than the group without ADHD (*p* = 0.004).

Table 2 shows the alcohol and cannabis use for the sample. Data were normally distributed.

**TABLE 2:** Alcohol and Cannabis use.

Substances used	SUD+ADHD *N* = 52 (29%)								SUD-ADHD *N* = 128 (71%)										
	Males				Females				Males				Females				Group comparison		Post-hoc
	*N*	%	M	s.d.	*n*	%	M	s.d.	*n*	%	M	s.d.	*n*	%	M	s.d.	*p*	*ƞ*_p_^2^	*p*
Alcohol use	23	44.2	-	-	14	26.9	-	-	61	47.7	-	-	18	14.1	-	-	0.62	0.02	-
Frequency of use	-	-	2.70	0.56	-	-	2.43	0.51	-	-	2.61	0.61	-	-	2.56	0.62	-	-	n/s
Age of onset	-		19.17	4.54	-	-	19.93	6.01	-	-	21.13	9.08	-	-	21.78	6.70	-	-	n/s
Years of consumption	-	-	12.61	8.22	-	-	13.00	6.82	-	-	13.48	10.00	-	-	8.83	5.09	-	-	n/s
Cannabis use	23	44.2	-	-	11	21.2	-	-	48	37.5	-	-	9	7.0	-	-	0.04*	0.09	-
Frequency of use	-	-	2.48	0.79	-	-	2.36	0.67	-	-	2.65	0.56	-	-	2.44	0.88	-	-	n/s
Age of onset	-	-	16.87	3.91	-	-	15.09	1.87	-	-	16.15	3.08	-	-	20.22	7.38	-	-	< 0.05†
Years of consumption	-	-	8.74	5.07	-	-	15.00	7.46	-	-	9.60	6.16	-	-	6.89	4.88	-	-	< 0.05‡

SUD, Substance use disorder; ADHD, Attention-deficit/hyperactivity disorder.

† , difference between females only;

‡ , ADHD females differed significantly from all other groups.

### Alcohol use

There were no significant differences for the use of alcohol neither between the SUD+ADHD and SUD-ADHD groups: *F*(3, 110) = 0.59, *p* = 0.62, *ƞ*_*p*_^2^ = 0.02 nor between the genders: *F*(3, 110) = 0.90, *p* = 0.44, *ƞ*_*p*_^2^ = 0.04.

Therefore, there were no significant differences in the frequency of alcohol use, start age and the duration of use between the SUD+ADHD and SUD-ADHD groups. Gender did not influence the outcome.

### Cannabis use

There was a significant main effect when the two SUD groups were compared. The SUD+ADHD group showed increased use of cannabis when compared with the SUD-ADHD group: *F*(3, 85) = 4.72, *p* = 0.004, *ƞ*_*p*_^2^ = 0.09. There was no main effect of gender but the analysis showed an interaction effect of gender on the SUD group: *F*(3, 85) = 4.72, *p* = 0.004, *ƞ*_*p*_^2^ = 0.14.

*Post-hoc* analysis (Bonferroni) showed that there was no difference in the frequency of cannabis use between the two groups for both genders but there was a significant difference between the females in the SUD+ADHD and SUD-ADHD groups for the age of onset of cannabis use (*p* = 0.02) with the ADHD females starting at an earlier age (M = 15.09, s.d. = 1.87) than the female SUD group without ADHD (M = 20.22, s.d. =7.38). There were no significant differences between the SUD+ADHD and the SUD-ADHD males and the ADHD females for the start age of cannabis consumption.

The SUD+ADHD females also used cannabis for a longer period of time (M = 15.00, s.d. = 7.46) not only than the SUD-ADHD females (M = 6.89, s.d. = 4.88, *p* = 0.02) but also than the SUD+ADHD (M = 8.74, s.d. = 5.07, *p* = 0.04) and SUD-ADHD (M = 9.60, s.d. = 6.16, *p* = 0.04) males.

## Discussion

Our study compared alcohol and cannabis use in treatment-seeking patients with and without comorbid ADHD.

To our knowledge, this is the first study that assessed alcohol and cannabis use in a South African sample of SUD patients with comorbid ADHD. Consumption-related variables and clinical factors were compared. Our results indicated a high incidence of ADHD symptomology amongst SUD patients (29%). This observation corresponds with previous studies in this field^10,51^ and can be explained by several causal factors, for example, genetic, neurobiological and psychosocial factors.^52^ No differences were found in alcohol use. However, significant differences were observed amongst the groups regarding cannabis consumption.

Contrary to expectations, we found no evidence of increased alcohol consumption, frequency of consumption, start age and the duration of use and individuals with SUD+ADHD. Neither between male or female nor between SUD+ADHD/SUD-ADHD. Several studies have positively correlated ADHD with increased alcohol abuse.^11,53^ The comparison group was not a typical sample of non-ADHD as they were also treated for alcohol use disorder. In addition, the small sample and high alcohol consumption in the South African population^31^ may have affected the results.

The current results suggest that also in the South African context, ADHD is highly prevalent in patients seeking treatment for cannabis use. When compared with the SUD-ADHD group, the SUD+ADHD group showed higher levels of cannabis use. Our results are consistent with other studies that describe similarly increased rates of cannabis use in patients with SUD+ADHD.^19,21,51^ The prevalence of ADHD in adults seeking treatment for cannabis use is estimated to be from 34% to 46%.^54^ Initially factors such as high novelty-seeking traits and self-medication for ADHD symptoms^55^ were thought to increase the risk of developing SUD in people with ADHD. However, recent findings by Petker et al. provide support for a link between cannabis use with immediate reward preference and symptoms of hyperactive-impulsive ADHD in young adults.^56^ The evidence for behaviour disinhibition is strengthened by the fact that ADHD shares genetic overlaps with novelty-seeking and substance abuse.^16^ Although cannabis appears to be a less spontaneous form of self-medication because its pharmacological mechanism has not yet been linked to the forebrain dopaminergic regulation system, animal models and electroencephalogram (EEG) studies have shown that cannabinoids affect impulsiveness and behavioural disinhibition.^54^ The relationship between cannabis use and ADHD remains unclear and more empirical studies are needed to understand the mechanisms that determine this comorbidity.

Furthermore, our research results show that, although there is no difference in frequency of use, female SUD+ADHD cannabis users show significantly different cannabis use patterns compared to female SUD-ADHD and SUD men with and without ADHD. Our results show that SUD+ADHD females have a significantly earlier age of onset of cannabis use than the SUD-ADHD females. Few previous studies have investigated gender differences in the relationship between ADHD and cannabis use.^37,57^ Compared to men with ADHD, women with ADHD, portrayed a more complex presentation, more anxiety, more sleep problems and perceived mental health impairment.^58,59^ Precocious puberty in girls is associated with increased internalising symptoms, such as depression and anxiety, during adolescence and in young adulthood,^60^ suggesting the possibility of self-medication to cope with their psychological distress. There appears to be a link between early puberty in females with ADHD and the development of SUD,^61^ which may explain the significant earlier use of cannabis amongst females within the SUD+ADHD group.

The results of our study also demonstrate that SUD+ADHD females significantly used cannabis for a longer period when compared not only with the SUD-ADHD females but also with SUD+ADHD and the SUD-ADHD males. Both research and clinical experience have shown that, compared with men, women with ADHD are more likely to experience anxiety and depression concurrently, and these secondary internalising disorders are more likely to be diagnosed rather than underlying ADHD.^62^ A study by Rucklidge and Tannock showed that adolescent women with ADHD have more psychological distress than men with ADHD.^62^ Also, compared to women without ADHD, women with ADHD have twice the prevalence of insomnia, anxiety, drug abuse, current smoking and depression.^63^ Kozak et al. found that the association between anxiety disorders and cannabis use might indicate that mental illness inclusive of ADHD can contribute to the increased likelihood of cannabis use – indicating that to cope with stressors and anxiety, females are more vulnerable to self-medicate with cannabis and are susceptible to developing and maintaining cannabis use.^64,65^

## Study strengths and limitations

Given the limited data on ADHD from South Africa, our study provides evidence of significant cannabis use, especially in females with ADHD, in treatment-seeking facilities in South Africa. A comparison of the employment status showed no significant difference, but the ADHD group showed a significantly higher level of education than the group without ADHD. There was a significant difference in ethnicity and home language, with the white Afrikaans speaking group being overrepresented in the SUD+ADHD group. We can speculate about the reasons for the observations, but few studies explored cultural factors in an ADHD diagnosis.^39,66^ Like other mental health disorders, social and cultural perspectives contribute to understanding adult ADHD and may play a significant role in diagnosing and accepting the condition.^67^

Treatment facilities are mainly located in Gauteng, North West and East Cape. As a result of insufficient rehabilitation facilities in South Africa, most provinces rely on referrals to areas where treatment centres are available. We did not find a significant link between increased alcohol consumption amongst individuals with SUD+ADHD. In a normal population, the difference might be significant, but in this small comparison treatment-seeking sample, all participants consumed alcohol. Patients were not diagnosed with cannabis use disorder (CUD), but were seeking treatment for SUDs and indicated significant cannabis use.

Finally, the data provided is cross-sectional; therefore, no causality can be inferred. It does not provide information on the relationship between ADHD symptoms, ADHD presentations or other comorbidities and the risk of substance use.

## Implications or recommendations

Given the specific ADHD symptoms and co-occurring disorders affecting clinical and population samples of ADHD,^7,54^ their role in the causal effects of ADHD on lifelong cannabis use deserves further study.

## Conclusion

Regardless of the limitations of the research, our research detected significant cannabis use in SUD patients with ADHD when compared with cannabis use in patients without ADHD. Amongst patients seeking treatment in South Africa between 2000 and 2013, an increase in the use of cannabis was observed. Consistent with previous studies,^21,22^ our cross-sectional data indicate increased levels of cannabis consumption amongst SUD+ADHD individuals, especially amongst SUD+ADHD females, highlighting the importance of women-specific treatment programmes to encourage utilisation of substance abuse treatment services.

These results strengthen the need to consider cannabis abuse in the context of ADHD in clinical interventions. South Africa needs to strengthen prevention and intervention activities against alcohol and cannabis use, especially in identified risk groups, like ADHD. Our findings, therefore, emphasise the need for early detection, diagnosis and treatment of ADHD to prevent substance use before it escalates to problematic levels.
